# Identification of transcriptional level variations in microRNA-221 and microRNA-222 as alternate players in the thyroid cancer tumor microenvironment

**DOI:** 10.1038/s41598-023-42941-1

**Published:** 2023-09-22

**Authors:** Rashida Khan, Aayesha Riaz, Samina Asghar Abbasi, Tanzeela Sadaf, Ruqia Mehmood Baig, Qaisar Mansoor

**Affiliations:** 1 Department of Zoology, PMAS-Arid Agriculture University, Rawalpindi, Pakistan; 2 Institute of Biomedical and Genetic Engineering (IBGE), Islamabad, Pakistan

**Keywords:** Biochemistry, Cancer, Cell biology, Genetics, Molecular biology, Biomarkers, Diseases, Health care, Medical research, Molecular medicine, Oncology, Risk factors

## Abstract

Thyroid cancer (TC) is caused by genetic factors and or their cross talk with lifestyle and environment. An important role of miRNA involvement has been identified in different human diseases alongside the cancer. The growing cloud of miRNA discoveries narrates miRNA-221 and miRNA-222 as key elements of ready arsenal in the cancer micro-niches. The aim of present study was to identify the variations of miRNA-221 and miRNA-222 expression in TC tissues and their likely association with TC. miRNA-221 and miRNA-222 were investigated for their expressional alterations in TC tissue samples and healthy thyroid tissue. Expression of miRNA-221 and -222 was analyzed through real time PCR. The relative gene expression of both the miRNA was quantified and statistically evaluated. miRNA-221 and miRNA-222 were found to be highly over expressed when compared with samples of multinodular goiter (MNG) and normal controls. Interestingly, it was also noted that miRNA-221 and miRNA-222 expression is working in a cluster in thyroid cancer patients. So, it can be concluded that the expressional alterations of miRNA-221 and -222 are playing their potential role in the development of thyroid cancer.

## Introduction

The endocrine related malignancy of thyroid which is recently found to be increasing rapidly from last few decades is considered to be thyroid cancer as its prevalence in many populations is increased. Differentiated thyroid carcinoma which constitute about 90% of thyroid cancers includes papillary thyroid cancer (PTC) and follicular thyroid cancer (FTC)^1^. Papillary thyroid carcinoma (PTC) is the most common type of thyroid cancer (85–90%)^2^. microRNAs (miRNAs) found endogenously and are single-stranded, noncoding 18- to 25-nucleotide RNAs which are playing their role in various biological and pathological processes of cell proliferation, differentiation and apoptosis^3^. miRNAs are contributing in the development of cancer in two ways. First, there may be the up regulation of some miRNAs which resulted in the silencing of tumor suppressor genes. Secondly there may be the down regulation of miRNAs which could resulted in more expression of oncogenes. So, the deregulations of miRNAs may lead to the over expression of some oncogenes or decreased expression of tumor suppressor genes leading to cancer^4^. Recent studies have also observed several miRNAs that are transcriptionally deregulated in papillary thyroid cancer (PTC) tissues in comparison with benign thyroid nodules and normal thyroid tissues^5^. miRNA-221 and miRNA-222 are important microRNAs that are abnormally expressed in thyroid cancer tissues compared with healthy controls^6^. Blocking miRNA-221 function by antisense methodology led to reduced cell growth of a human PTC cell line, while its overexpression led to increased colony formation, indicating a critical role of miRNA-221 in thyroid carcinogenesis^7^. Studies demonstrated that miRNA-221 and miRNA-222 are transcriptionally up-regulated in PTC tumors in comparison with normal thyroid tissue^8^. A functional study showed that miRNA-221 and miRNA-222 are endogenous regulators of P27^Kip1^ protein expression, which represents a very important regulator of the cell cycle^9^. With the up-regulation of these miRNAs, there is a dramatic loss of KIT transcript and Kit protein, both of which are involved in the pathogenesis of thyroid cancer.

In Hashimoto’s Thyroiditis small extracellular vesicle (sEV) mediated miR-142-3p is a communication mode between thyrocyte cells and T-lymphocytes favoring the progression of the disease^10^.

It is confirmed that the down-regulation of HOXB5 by endogenous or exogenous miRNA-221 involves the progression of PTC^11^. It is investigated that expression of miRNA-221 and miRNA-222 had 5- to a 35-fold differential in FNAB samples of PTCs compared with other thyroid nodules^7^. It was also observed that the overexpression of miRNA-221, and miRNA-222 were significantly associated with extrathyroidal invasion in PTCs^12^. In the current study, aberrant expression of these miRNAs in thyroid cancer tissue samples playing their potential role in the development and progression of PTC was investigated.

Overexpressed miRNA-221 and miRNA-222 in PTC are closely correlated with clinical and pathological characteristics^13^. Overexpression of miRNA-221 in PTC samples was considered a potential biomarker for PTC recurrence and excessively secreted miRNA-221 levels have been observed in PTC, follicular and anaplastic thyroid cancers^9^. Previous studies also indicated that overexpression of miRNA-221 played an important role in the proliferation of thyroid cancer cells^14^. All these findings strongly suggest a close correlation between miRNA-221and miRNA-222 and the development of PTC. By keeping this in view the present study was conducted to check the expression of miRNA-221 and miRNA-222 in thyroid cancer patients and multinodular goiter subjects along with their adjacent normal tissue samples. This study aimed to check whether these miRNAs are overexpressed in the studied population and have a risk associated with the development of thyroid cancer in the Pakistani population like other populations.

## Material and methods

### Sampling and data collection

Fresh tissue samples were taken in falcon tubes having RNA later and immediately stored at 4 °C. Trizol method of RNA extraction was used to extract the RNA from tissue samples. RNA quantification was done by Nano drop to check the concentration of RNA. After extraction of RNA, cDNA was synthesized by using a commercially available kit according to manufacturer protocol. Synthesized cDNA was used and RT-PCR was carried out for the expressional analysis of miRNA-221 and miRNA-222.

Thyroid stimulating hormone (TSH), Thyroglobulin (TG), Antithyroglobulin (ATG) antibodies, and tumor grades were recorded on a questionnaire specially designed for the study. The study protocol was approved by the ethical review committee of PMAS Arid Agriculture University Rawalpindi and from the hospitals of Rawalpindi and Islamabad Pakistan.

### Expressional analysis

The subjects were studied for expressional analysis of miRNA-221 and miRNA-222. Briefly, cDNA was prepared and real time PCR was performed for the cDNA samples using specific primers for specified miRNAs. The primers, F-5′-GCATGAACCTGGCATACA-3′-F and reverse R-5′-AGCAGACAATGTAGCTGTTG-3′-R were selected for miRNA-221. Similarly, the primers F-5′-AAGGTGTAGGTACCCTCAAT-3′-F and reverse R-5′-CCAGATGTAGCTGCTGATTAC-3′-R were selected for miRNA-222 for their expressional analysis. RT-PCR was carried out in a total volume of 25 µl in each PCR tube. It includes 0.5 µl of 100 pmol/µl of each of forward and reverse primers, 13 µl of SYBR Green PCR Master Mix, 2 µl cDNA template RT+/RT−/water/patient sample/control and 9 µl of PCR water.

### Statistical analysis

The results were statistically calculated and analyzed using SPSS (version 20) and GraphPad Prism version 9.5 for Mac, GraphPad Software, http://www.graphpad.com. The statistical tests; T-test, Chi-square test for p-value calculation for the comparison of gene expression among thyroid cancer patients, MNG and controls were conducted.

### Use of human participants

The study protocol for the induction of human participants in the study was approved by the ethical review committee of PMAS Arid Agriculture University Rawalpindi Pakistan on October 03, 2018 and confirmed that all research will be performed in accordance with relevant guidelines/regulations, and it is confirmed that informed consent was obtained from all participants and/or their legal guardians. In addition, the research involving human participants will be performed in accordance with the Declaration of Helsinki.

## Results

### Demographic and clinical data

The subjects included 30 thyroid cancer patients along with 30 adjacent normal tissue samples of the same patient and 30 multinodular goiter patients along with their normal thyroid tissue. Patients were categorized into three different age groups i.e. later age (more than 60), middle-age (40–60), and early age (below 40). 34% patients in early age, 51.1% in middle and 14.9% patients in the later age group were present. It was seen that all the patients were having papillary thyroid cancer with its different subtypes. Age, gender and metastatic status of the patients was also recorded (Table 1).

**Table 1:** Clinical hallmarks of thyroid cancer patients.

Variables	Patients	Frequency	Comparative data
Age	< 50 (years)	34.78	± 45.6(years) [27]
	50–60 (years)	51.97	
	> 60 (years)	15.59	
Gender	Females	86.4	
	Males	14.6	
Metastasis	Metastatic	6.41	0.7% [28]
	Non-metastatic	93.53	

**Figure 1:**
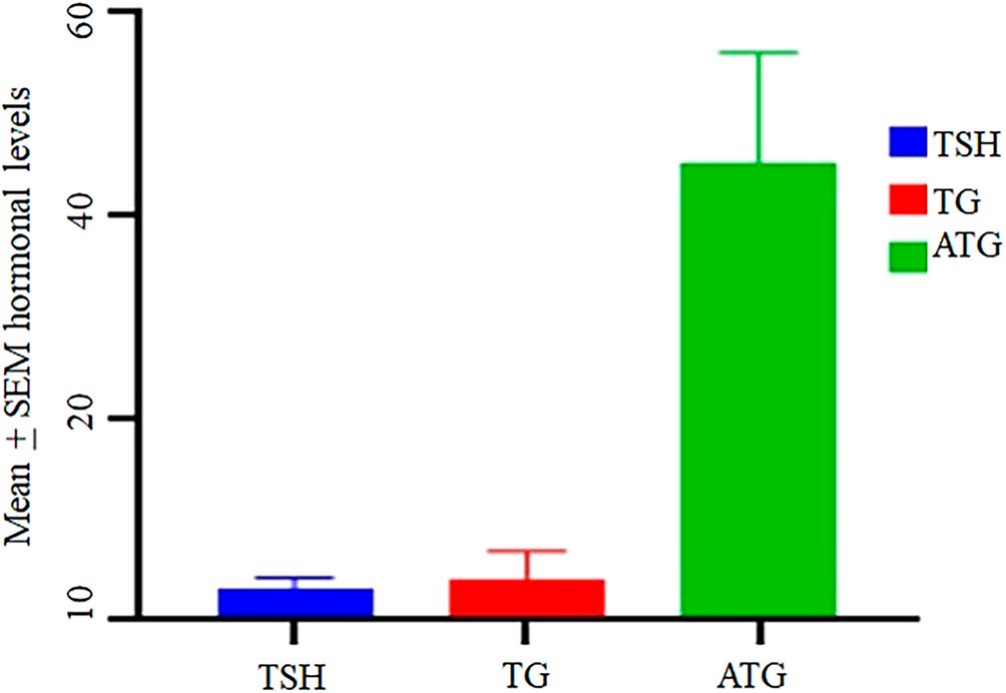
Hormonal levels TSH, TG and ATG in thyroid cancer patients. The mean values of the levels were calculated with standard error of the mean (± SEM). SEM error bars are shown on the top of each bar on the chart.

**Figure 2:**
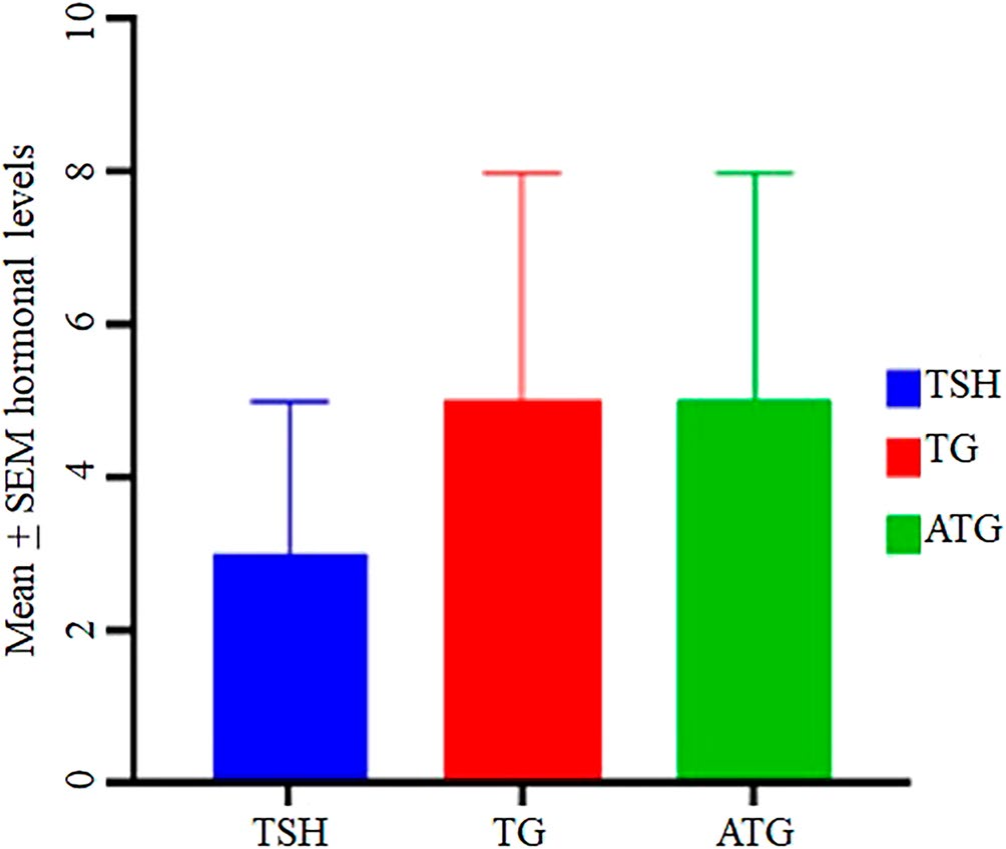
Hormonal levels TSH, TG and ATG in MNG patients. The mean values of the levels were calculated with standard error of the mean (± SEM). SEM error bars are shown on the top of each bar on the chart.

TSH, TG, ATG, and tumor grades were also recorded for the thyroid cancer patient group as well as for MNG patient group (Figs. 1 and 2).

### Analysis of expression of *miRNA-221* and *miRNA-222*

#### Expression of miRNA-221 and thyroid cancer

**Figure 3:**
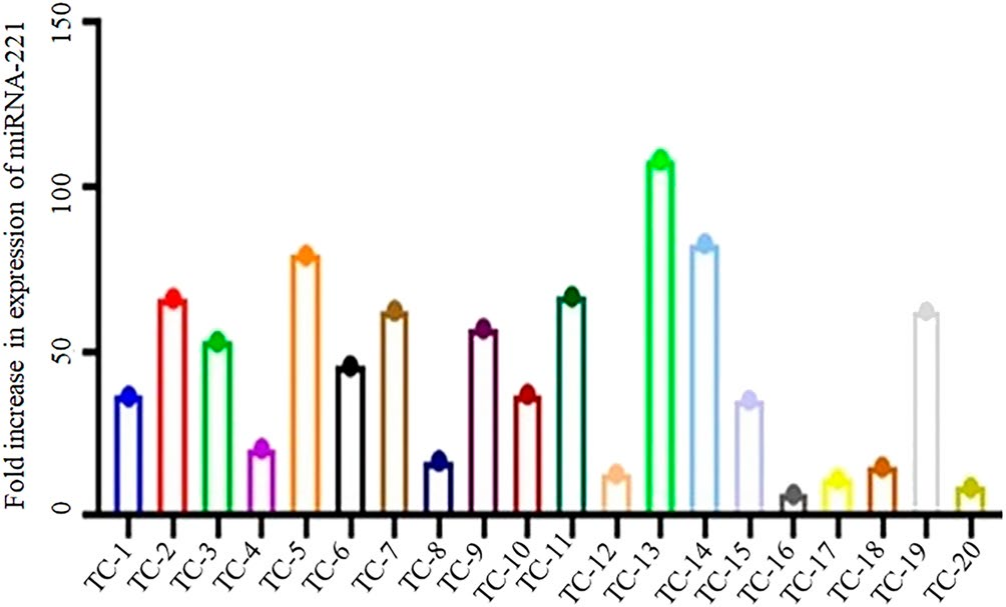
Fold increase in expression of miRNA-221 in thyroid cancer patients (TC-1 to TC-20). The fold increase in the expression was determined by relative gene expression through real time PCR.

**Figure 4:**
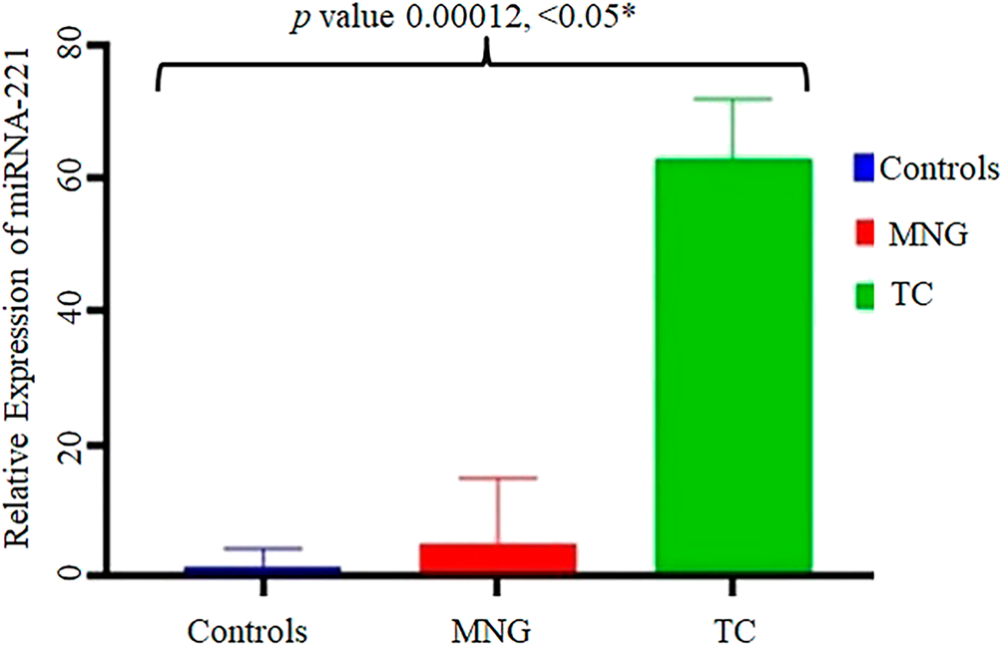
Relative expression of miRNA-221 in thyroid cancer patients, MNG and normal controls. ANOVA test was applied to thyroid cancer, MNG and controls. *p value determined by comparison of the relative gene expression observed in the three groups i.e. Controls, MNG and TC in ANOVA test.

In the present study 30 thyroid cancer tissue samples were checked for their expression (Fig. 3) and then the expression was compared with 30 MNG tissue samples along with their adjacent normal control samples in the Pakistani population. The expression of miRNA-221 was found to be significantly higher (*p* value 0.00012) in thyroid cancer samples when its expression was compared through One way ANOVA with the patients of MNG and normal controls (Fig. 4). The ANOVA was followed by Post Hoc Tukey’s test showing multiple comparison among controls, MNGs and thyroid cancer patients (Table 2).

**Table 2:** miRNA-221 expression analysis by (a) one way ANOVA followed by (b) Post hoc test multiple comparisons for Tukey’s HSD.

(a)						
Groups	Sum of squares	df	Mean square	F	Significance	
Between groups	22,821.606	2	11,410.803	40.984	0.00012*	
Within groups	15,870.012	57	278.421			
Total	38,691.618	59				

(b)						
Group A	Group B	Mean difference (A-B)	Std. error	Significance	95% confidence interval	
					Lower bound	Upper bound
Control	MNG	− 3.64700	5.27656	0.770	− 16.3446	9.0506
	Thyroid Cancer Patients	− 43.07450**	5.27656	0.000	− 55.7721	− 30.3769
MNG	Control	3.64700	5.27656	0.770	− 9.0506	16.3446
	Thyroid Cancer Patients	− 39.42750**	5.27656	0.000	− 52.1251	− 26.7299
Thyroid Cancer Patients	Control	43.07450**	5.27656	0.000	30.3769	55.7721
	MNG	39.42750**	5.27656	0.000	26.7299	52.1251

*Significance at the 0.05 level.

**The mean difference is significant at the 0.05 level.

#### Expression of miRNA-222 and thyroid cancer

**Figure 5:**
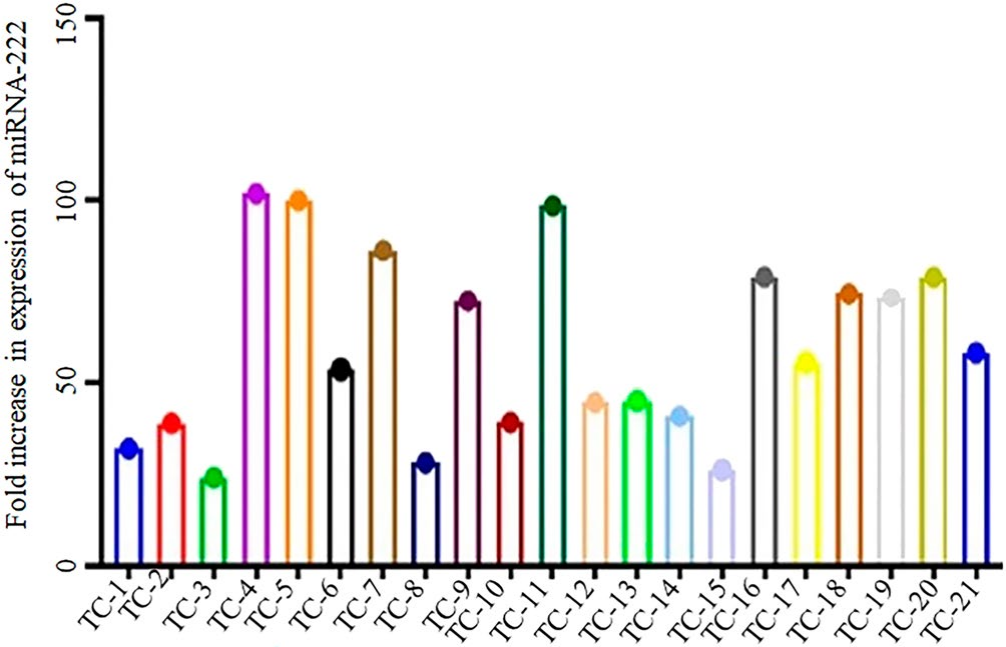
Fold increase in expression of miRNA-222 in thyroid cancer patients (TC-1 to TC-21). The fold increase in the expression was determined by relative gene expression through real time PCR.

**Figure 6:**
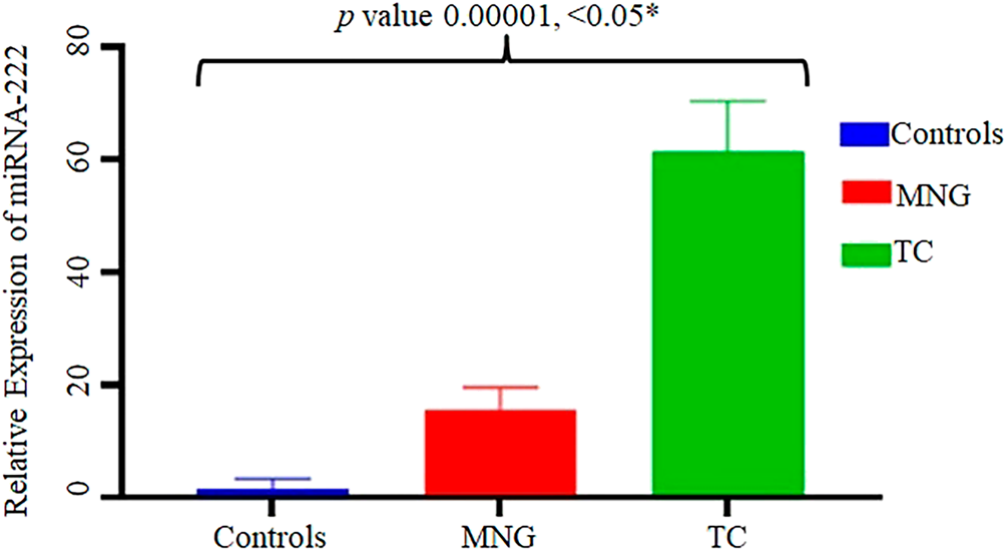
Relative expression of miRNA-222 in thyroid patients compared to MNG and normal controls. ANOVA test was applied to thyroid cancer, MNG and controls. *p value determined by comparison of the relative gene expression observed in the three groups i.e. Controls, MNG and TC in ANOVA test.

It was observed in this study that the expression of miRNA-222 was significantly high (*p* value 0.00001) as revealed by One way ANOVA in thyroid cancer samples while the expression was quite low in MNG and normal control samples (Figs. 5 and 6). The ANOVA test for the multiple comparisons among controls, MNGs and thyroid cancer patients was followed by Post Hoc Tukey’s test (Table 3).

**Table 3:** miRNA-222 expression analysis by (a) one way ANOVA followed by (b) post hoc test multiple comparisons for Tukey’s HSD.

(a)						
Groups	Sum of squares	df	Mean square	F	Significance	
Between groups	40,662.521	2	20,331.260	89.587	0.00001*	
Within groups	12,935.862	57	226.945			
Total	53,598.382	59				

(b)						
Group A	Group B	Mean difference (A-B)	Std. error	Significance	95% confidence interval	
					Lower bound	Upper bound
Control	MNG	− 7.49700	4.76387	0.265	− 18.9609	3.9669
	Thyroid cancer patients	− 58.5895**	4.76387	0.000	− 70.0534	− 47.1256
MNG	Control	7.49700	4.76387	0.265	− 3.9669	18.9609
	Thyroid cancer patients	− 51.09250**	4.76387	0.000	− 62.5564	− 39.6286
Thyroid cancer patients	Control	58.58950**	4.76387	0.000	47.1256	70.0534
	MNG	51.09250**	4.76387	0.000	39.6286	62.5564

*Significance at the 0.05 level.

**The mean difference is significant at the 0.05 level.

#### miRNA-221 and miRNA-222 working in a cluster in thyroid cancer

**Figure 7:**
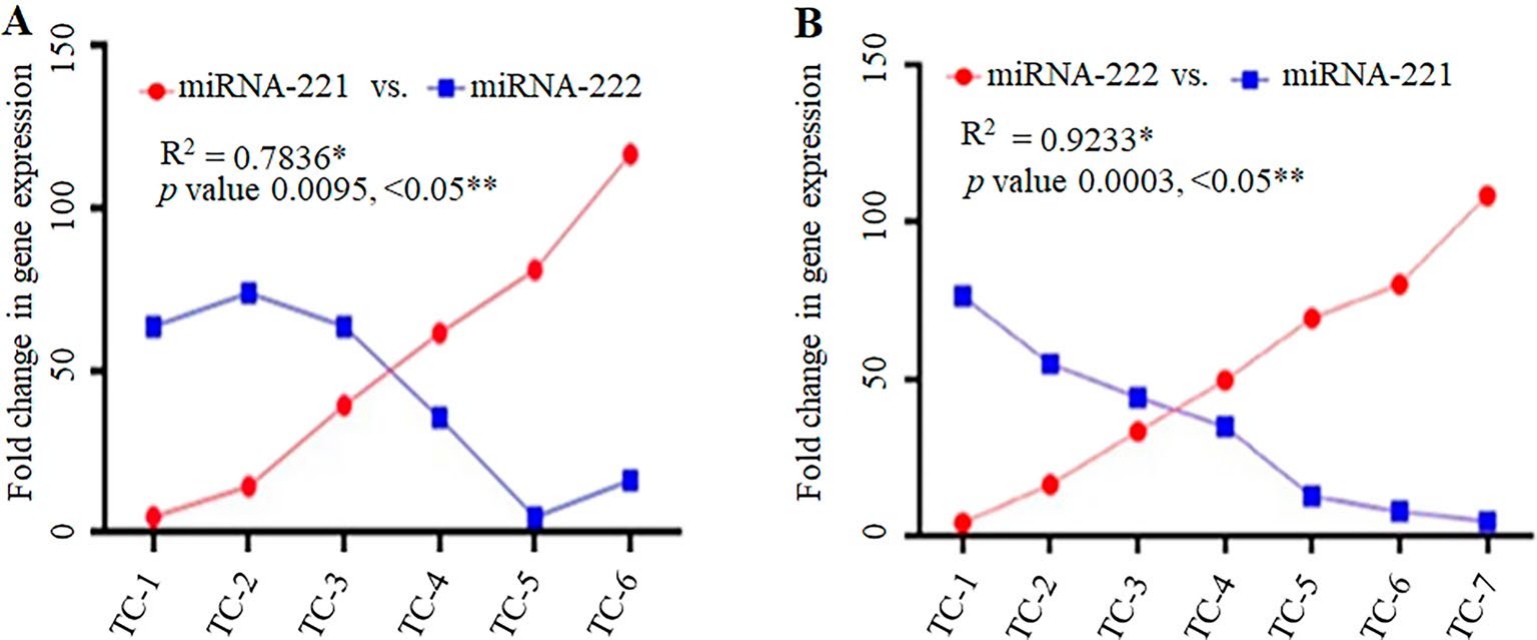
Clustered fold change in the expression of miRNA-221 and miRNA-222 in thyroid cancer patients (**A**) TC-1 to TC-6, (**B**) TC-1 to TC-7. The clustered or combined fold change in the gene expression was calculated by correlation of the data test. *R-squared (R^2^) and **p values are represented from the correlation of the data test.

In the present study, it was demonstrated that both miRNA-221 and miRNA-222 showed their expression in a cluster, and also, they are compensating each other’s expression in cancer progression. It was seen that in the same patient if the expression of miRNA-221 was low the expression of miRNA-222 was very high to continue the cell proliferation of cancerous cells (Fig. 7A). In the same way when the expression of miRNA-222 was low in some patients the expression of miRNA-221 was very high to compensate for the miRNA-222 to continue the cell growth and proliferation to increase the progression of cancerous cells (Fig. 7B).

## Discussion

miRNAs are potential biomarkers for the detection of cancer, and numerous studies have been performed to determine the relationship between miRNAs and thyroid cancer. The relative gene expression of miRNA-221 and miRNA-222 was investigated in TC, MNG and normal controls. The target genes of miR-222 and miR-221 are p27 and p57 and they may interfere essentially to onset thyroid oncogenesis^9^. Additionally, miRNA-221 and miRNA-222 were found at the highest levels in BRAF- and RAS-positive PTCs^15^. Moreover, the up regulation of miRNA-221 and miRNA-222 may not only be involved in the development of PTC but also in invasion and metastasis of PTC^11^.

The results of present study showed significantly high expression of the miRNAs in thyroid cancer patients than MNG and normal samples. Strong evidence of up-regulated expression of miRNA genes i.e. miRNA-221 and 222 in PTC and an up to 70-fold boosted expression of miRNA-221in PTC compared to negligible expression in the healthy thyroid tissues has been reported^6,16^. Additionally, miRNA-222 and miR-146b have been shown tenfolds up-regulated in classic variants of PTC in contrast to normal thyroid tissue^17^.

Additionally miRNA-221 over expression has been identified in clear cell renal cell carcinoma^18^. The up regulation of miRNA-221 and miRNA-222 has been reported in breast neoplasm, glioblastomas, and lung and hepatocellular carcinoma^19^ and significant over expression associated with extra-thyroidal invasion in PTCS^11^. Moreover miRNA-221 was the most reported as the most favorable miRNA in differentiating benign from malignant thyroid pathology^20^. These gene have been shown to modulate cell proliferation, telomere activity, apoptosis, tumor suppressors, angiogenesis, and autophagy^21^.

The present study remarkably showed clustered working of the miRNA-221 and miRNA-222 in TC. This can be explained as if expression of one of the miRNAs was lower in a any of the patient, the expression of the other miRNA was found up-regulated in the same patient. This clustered mechanism in miRNAs expression indicates their certainly crucial role in the TC. The amalgam of scientific literature comprehensively suggests combined involvement of miRNA221- and miRNA-222 together. But surprisingly findings of present study represented an unidentified expressional regulation of these miRNAs in thyroid cancer in alternate manner i.e. one of the miRNA if down-regulated the other one’s expression is boosted. This leads to the concept that there is a dire augmented back-to-back support and or involvement of this miRNA221 and miRNA-222 combo.

## Conclusion

The current study revealed that expression of both miRNA-221 and miRNA-222 is working in an alternate clustered fashion in TC tumor. Since this effect is first of its kind at the expression level in the tumor microenvironment the study regards and proposes it as *“Khan’s microRNAs clustering in thyroid cancer tumor”*. This specific miRNA signature composed of miR-221, and miR-222 could serve as potential clinical biomarker as therapeutic target in TC patients, particularly in PTC. These findings showing up regulation of miRNA-221 and miRNA-222 might be helpful and facilitate the surgical management of thyroid cancer patients and can be integrated into the prognostication system of thyroid cancer in near future.

## Data Availability

The data related to this study is included in the manuscript. The first author RK can be contacted at the email address rashidakhan3919@gmail.com for the availability of any data related to this study.
